# Validity and reliability of the Smoking-related Health Literacy Assessment Scale

**DOI:** 10.31744/einstein_journal/2022AO5156

**Published:** 2022-02-03

**Authors:** Adélia Dayane Guimarães Fonseca, Rene Ferreira da Silva, Claudiana Donato Bauman, Liliane Lacerda Silva, Carla Silvana de Oliveira e Silva, Andrea Maria Eleutério de Barros Lima Martins

**Affiliations:** 1 Universidade Estadual de Montes Claros Montes Claros MG Brazil Universidade Estadual de Montes Claros, Montes Claros, MG, Brazil.

**Keywords:** Health literacy, Tobacco use disorder, Tobacco, Validation study, Reproducibility of results

## Abstract

**Objective:**

To create an instrument to assess smoking-related health literacy among smokers, and to estimate validity of its content and reliability.

**Methods:**

A methodological, quantitative study. The creation of the instrument included the following steps: establishing a conceptual structure; defining objectives and target population; preparation of items or response scales; selecting and organizing items; instrument structuring; content validation and pre-test. The instrument was named Smoking-related Health Literacy Assessment Scale.

**Results:**

The Smoking-related Health Literacy Scale had statistically significant measures of validity and reliability. Test-retest revealed substantial to almost perfect levels of reliability (reproducibility).

**Conclusion:**

The Smoking-related Health Literacy Scale can provide researchers with a valid and statistically significant instrument, regarding content validity and reliability.

## INTRODUCTION

Smoking is a significant risk factor for chronic noncommunicable diseases (NCD), with higher mortality, including cancer, chronic respiratory and cardiovascular diseases, and diabetes.^(1-3)^ Chronic NCD account for more than half of deaths worldwide and for most deaths reported in Brazil.^(4-6)^ The smoking epidemic expands beyond borders and is a global public health concern, and a major known cause of preventable morbidity and mortality.^(7,8)^

According to the World Health Organization (WHO), 6 million deaths per year are caused by active or passive smoking.^(9)^ Also, the Brazilian Public Health System (SUS - *Sistema Único de Saúde*) hospitalization and chemotherapy procedure costs attributable to smoking among individuals aged 35 years or older, in 2005, amounted to R$ 338,692.516,02 - approximately one third of overall SUS costs.^(6)^

In this context, smoking has been attracting increasing attention in research globally. Smoking prevalence, the profile of smokers and their practices, motivations for smoking cessation or continuation, and addiction-related factors are major focuses of investigation. The motivation to understand smoking encourages investigations into the topic, including health literacy, since is thought to be associated with health outcomes and is, therefore, a matter of growing concern among researchers and professionals in this field. Health literacy includes personal, cognitive, and social skills associated with the ability to access, understand, evaluate and use health-related information, which is required to promote and/or maintain good health. It also involves individual and contextual determinants, such as patient-health care professional communication skills, cultural aspects, health system complexities, and demands driven by different scenarios or contexts.^(10-12)^

The current dynamics of interest for health-related issues and respective outcomes has led researchers and international organizations to develop instruments with applications, aimed to assess and/or measure health-related events.^(12)^

There is a growing number of assessment scales and/or questionnaires. However, the quality of many of these instruments was incompletely and/or inappropriately examined.^(13,14)^ The usefulness of instruments and their ability to provide robust results from a scientific standpoint are directly related to their quality; *i.e*., their reliability, validity, responsiveness and interpretability.^(14)^ Hence, in 2006 and 2007, an international, multidisciplinary Delphi consensus study, addressing quantitative assessment of health-related states and events, was conducted by 43 experts to establish standards for examination of the methodological quality of studies investigating these events or states (*i.e*., the properties of instruments designed to measure health-related events). A checklist (COnsensus-based Standards for the selection of health Measurement INstruments - COSMIN) comprising consensus-based standards for post-assessment selection of health measurement instruments was created. COSMIN checklist includes a set of parameters across four domains: three for instrument assessment - reliability, validity, and responsiveness - and one for interpretability. Interpretability is not a measure *per se*. However, it is a significant factor in the assessment of instrument appropriateness for application in research and/or clinical practice.^(14)^

Questionnaires are ancillary instruments in clinical practice, and are also used in health assessment and scientific research in the field of public health. Hence, these instruments have a significant impact on care-, treatment- and/or intervention-related decisions, as well as on formulation of health programs and institutional policies.^(13)^ No instruments examining levels of health literacy about smoking have been found.

## OBJECTIVE

To create an instrument to assess smoking-related health literacy among smokers, and to estimate validity of its content and reliability.

## METHODS

A methodological, quantitative study conducted in 2016, with 62 participants registered at two *Estratégias Saúde da Família* (ESF) [Family Health Strategy program] units, in the city of Montes Claros, MG, Brazil.

This study included the following steps when creating health measurement instruments: establishing a conceptual structure; defining of objectives and target population; preparation of items or response scales; selecting and organizing items; instrument structuring, content validation and pre-test.^(12)^ First, an instrument aimed to measure health literacy about smoking named Smoking-related Health Literacy Scale (EAS-T) was created. Scale content validity and reliability (psychometric properties) were then examined.

The first step to create the instrument was a literature review, conducted to identify studies addressing “health literacy” and “smoking”, as well as the major measurement instruments used. This review was performed at MEDLINE^®^, PubMed^®^ and Virtual Health Library (VHL) databases. Publications in Portuguese, English and Spanish were retrieved. The purpose of the instrument to be created was defined: to assess health literacy about smoking. This step also included the definition of the target population: individuals aged 18 years or older registered at ESF located in the city of Montes Claros.

In the second step, items and the response scale were designed. These were carefully defined based on findings of the specific literature search on health literacy and Smoking, as well as on analysis of several pre-existing health literacy assessment instruments. With regard to theory, the theoretical model proposed by Sørensen et al.,^(15)^ was particularly emphasized, and some health literacy assessment instruments, such as the Short Assessment of Health Literacy for Portuguese-speaking Adults (SALPHA)^(16)^ selected for analysis.

Items included in the Smoking-related Health Literacy Assessment Instrument were methodically organized for general formatting purposes. The title, instructions and response scales were defined in this step of the process. The appropriateness and consistency of instrument items were examined using the content validation technique. At this stage, the instrument was submitted to expert judgment, with particular focus on quality of items, and their representativeness regarding the proposed objectives. The instrument was judged by ten health professionals, with different levels of education and expertise (undergraduate degree holders, specialists, family health residents, masters´ and PhD degree holders), who were invited by convenience, based on their respective professional activities and background.

Content validation was carried out as follows. Invited professionals agreed to participate as judges by signing a term of acceptance and authorization. Judges were duly informed about objectives, methods, and procedures of instrument creation. Each judge conducted an independent assessment prior to meetings with other panel members. Judges were then asked to judge items and determine their comprehensiveness. Judges determined whether concepts were duly covered by the set of items, whether the content was appropriate for the target population, whether domain structure and content were correct, and whether domain content was representative. In the third step examined instrument items for propriety and qualitative properties (objectivity, clarity, readability, ease of understanding, and relevance or representativeness).^(17,18)^

The above-mentioned procedures were conducted in an individual and independent manner, addressing the quantitative aspect of content assessment. Qualitative assessment was performed during meetings with judges and instrument creators. These meetings were guided by questions brought up over the course of item definition and selection, and involved different specialists, who were selected according to the nature of the piece of information. The fourth and last step involved content validation and consisted of discussion groups, including all judges to produce the final version of the instrument. Following conclusion and inclusion of all suggestions, the instrument was released and applied to a pilot sample comprising 60 individuals selected out of the target population.

Pilot study interviews were conducted by previously trained interviewers (community health workers and students of dentistry and physical education). Data were collected at the home of individuals registered in the ESF following awareness raising efforts and invitation by members of the ESF team. Upon completion of the pilot study, interviewers participated actively in discussions with judges, during which their impressions regarding the need of instrument modifications/adjustments were communicated.

Reliability assessment was based on test-retest and internal consistency. Test-retest was used to examine the ability of the test to produce identical results when measuring a given event in the same people on different occasions. Ideally, measures obtained on different occasions should be correlated.^(19)^

In order to determine the reliability of the instrument designed to assess smoking-related health literacy, the instrument was applied to a sample comprising 62 participants^(20)^ using the test-retest method. Selected participants were duly registered in two ESF units belonging to the Primary Health Care network of the municipality. *Estratégias Saúde da Família* units were selected at random and pilot study participants were excluded. Samples comprising a minimum of 50 and a maximum of 100 subjects are thought to be appropriate to examine the internal consistency and test-retest of measurement instruments.^(19,21)^

Participants were interviewed by the same interviewer on different occasions, 7 to 14 days apart. This sample comprised participants who signed an Informed Consent Form (ICF) and met the following criteria: 18 years of age or older, and registration at selected ESF units. Exclusion criteria were as follows: obvious signs of cognitive impairment and/or depression, illiteracy, non-native speaker of Portuguese, vision/hearing problems (reported or perceived), and/or intoxication by drugs or alcohol at the time of interview. Cognitive impairment assessment was based on the Mini Mental State Examination (MMSE).

Statistical analyses were performed using (SPSS) for Windows, version 20.0, and Excel software. Sample profile was described using absolute and relative frequencies (categorical variables) or mean, standard deviation (SD), median and maximum and minimum values (continuous variables).

Instrument internal consistency was tested using Cronbach’s alpha. This parameter is calculated to examine the internal consistency of items and ranges from zero to one. The closer to one, the greater the reliability of the assessment instrument. Values equal to or greater than 0.7 are considered acceptable.^(22)^ In the reliability and internal consistency study, the level of agreement between two independent observations was measured using test-retest. Given the binary nature of assertive responses in the Smoking-related Health Literacy Assessment Instrument, the Kappa coefficient was applied to each instrument item. Agreement was rated according to dedicated literature, as follows: Kappa <0.00, almost non-existent; 0 to 0.19, low; 0.20 to 0.39, unsatisfactory; 0.40 to 0.59, moderate; 0.60 to 0.79, substantial; and 0.80 to 1.00, almost perfect.^(23)^

This research project was approved by the Research Ethics Committee of *Universidade Estadual de Montes Claros* (Unimontes) and registered at the *Sistema Nacional de Informações sobre Ética em Pesquisas envolvendo Seres Humanos* (SISNEP), opinion no. 764.743, CAAE: 34687414.0.0000.5146.

## RESULTS

Most participants in this study (52 out of 62, 83.9%) were female. The mean age was 54.9±9.97 years (range 29 to 77 years). Level of education ranged from zero to 12 years or more of study (mean 5.63±3.99 years).

As to stratified age, a large proportion of participants (37, 59.7%) were aged 40 to 59 years. With regard to level of education measured in years of study, a significant proportion of participants (25, 40.3%) had between 1 and 4 years of study, whereas 19 (30.6%) and ten (16.1%) had from 5 to 8, and from 9 to 11 years of study respectively.^(24)^

As to occupation, most participants were housewives (42, 67.7%), retired (5, 8.1%), construction workers (3, 4.8%), teachers (2, 3.2%) or shop assistants (2, 3.2%). These data as shown in table 1.

**Table 1:** Sociodemographic data of individuals registered with *Estratégia Saúde da Família*

Variables	n (%)	95%CI
Sex		
Male	10 (16.1)	8.1-25.8
Female	52 (83.9)	74.2-91.9
Stratified age		
20-39	4 (6.5)	1.6-12.9
40-59	37 (59.7)	46.8-72.6
60-79	21 (33.9)	21.0-46.8
Level of education, years of study		
0	5 (8.1)	1.6-14.5
1-4	25 (40.3)	27.4-51.6
5-8	19 (30.6)	19.4-43.5
9-11	10 (16.1)	8.1-25.8
≥12 or more	3 (4.8)	0.0-9.7
Occupation		
Housewife	42 (67.7)	56.5-79.0
Retired	5 (8.1)	1.6-16.1
Recyclable material collector	1 (1.6)	0.0-4.8
Hairdresser	1 (1.6)	0.0-4.8
Teacher	2 (3.2)	0.0-8.1
Merchant	1 (1.6)	0.0-4.8
Rural worker	1 (1.6)	0.0-4.8
Construction worker	3 (4.8)	0.0-11.3
Micro entrepreneur	1 (1.6)	0.0-4.8
Dressmaker	1 (1.6)	0.0-4.8
Eyeglass maker	1 (1.6)	0.0-4.8
Shop assistant	2 (3.2)	0.0-8.1

95%CI: 95% confidence interval.

Content validation procedures, variables and items included in the final version of the Health Literacy Assessment Scale are shown in table 2.

**Table 2:** Final version of the Smoking-related Health Literacy Assessment Scale (EAS-T)

Primary word	Association word		
1. Tobacco	( ) Plant	( ) Object	( ) Do not know
2. Smoking	( ) Examination	( ) Habit	( ) Do not know
3. Passive smoking	( ) Medication	( ) Smoke	( ) Do not know
4. Cessation	( ) Stop	( ) Continue	( ) Do not know
5. Nicotine	( ) Exercise	( ) Cigarette	( ) Do not know
6. Addiction	( ) Dependence	( ) Freedom	( ) Do not know
7. Cardiovascular	( ) Lung and bones	( ) Heart and blood vessels	( ) Do not know
8. Cigar	( ) Chew	( ) Smoking	( ) Do not know
9. Chewing tobacco	( ) Sniff	( ) Drink	( ) Do not know
10. Emphysema	( ) Hand	( ) Lung	( ) Do not know
11. Osteoporosis	( ) Viral infection	( ) Bone	( ) Do not know
12. Lung	( ) Bronchitis	( ) Arthritis	( ) Do not know
13. Risk	( ) Danger	( ) Shelter	( ) Do not know
14. Inhale	( ) Breathe	( ) Walk	( ) Do not know
15. Mortality	( ) Death	( ) Birth	( ) Do not know
16. Tobacco user	( ) Artist	( ) Smoker	( ) Do not know
17. Impotence	( ) Desire	( ) Impossibility	( ) Do not know
18. Thrombosis	( ) Nails	( ) Veins	( ) Do not know

The reliability (reproducibility) of responses given to each word and/or term ranged from 0.75 to 1 (Kappa coefficient) (Table 3). The instrument had good internal consistency (Cronbach’s alpha, 0.915).

**Table 3:** Levels of agreement (Kappa coefficient) and internal consistency (Cronbach’s alpha) attributed to EAS-T words and/or terms

Word and/or term	Reproducibility Kappa*	Internal consistency Factor alpha, if item is deleted
Tobacco	0.869	0.909
Smoking	0.887	0.911
Passive smoking	0.746	0.910
Cessation	0.794	0.912
Nicotine	1.000	0.914
Addiction	1.000	0.916
Cardiovascular	1.000	0.911
Cigar	0.767	0.912
Chewing tobacco	0.910	0.916
Emphysema	0.961	0.911
Osteoporosis	1.000	0.916
Lung	1.000	0.916
Risk	1.000	0.916
Inhale	1.000	0.912
Mortality	1.000	0.915
Tobacco user	0.866	0.909
Impotence	0.917	0.913
Thrombosis	0.931	0.914

* p<0.05 for all items.

## DISCUSSION

Smoking is a major public health concern in Brazil from a morbidity, mortality and health care cost perspective.^(23)^ Smoking habit assessment provides insights into people’s relationships and life context. Likewise, collective action planning and implementation must be based on people’s actual opinions and behaviors. Hence, the selection of assessment instruments is a vital decision. Investigations in this field may support policies and therapeutic approaches designed to tackle this significant public health concern in the individual and the community level.^(10)^

Measurement of certain constructs requires valid and reliable instruments. The full development of a novel health measurement instrument is a complex procedure, demanding several resources and mobilization of abilities and knowledge in different areas.^(13,19)^

Population health assessment requires instruments capable of providing accurate, valid and interpretable data. Measurements must also provide robust results from a scientific standpoint. The significance of findings derived from such measurements is closely related to instrument reliability and validity. In spite of divergences, researchers believe reliability and validity to be the most important properties of measurement instruments.^(10,25,26)^

The assessment of psychometric properties of measurement instruments has been modified in the last century and trends pertaining to other contexts have been incorporated. According to such trends, the longer the instrument employed to assess a given construct, the better the validity. Therefore, a larger number of items would contribute to the calculation of reliability formulas, such as Cronbach’s alpha. However, in the last decades, this concept has been modified by a theoretical line of thought known as “item response theory” (IRT), which suggests shorter scales are as reliable as long ones.^(27)^

With regard to reliability, high internal consistency was detected in this study. Internal consistency can be defined as the ability to determine the correlation or homogeneity between items in a scale type instrument (*i.e.*, whether the instrument actually measures the intended theoretical construct). Cronbach’s alpha is an expressive and desirable measure of internal consistency for instruments aimed to measure a single construct using multiple items. It measures the correlation between questionnaire responses according to the profile of responses given by respondents, and therefore represents a mean correlation between questions. Ideally, the value should fall between 0.70 and 0.95.^(19,27)^ Cronbach’s alpha values in this study were higher than 0.90, indicating very high internal consistency. The exclusion of four items in the scale (addiction, chewing tobacco, osteoporosis, lung and risk) led to a slight increase in the alpha factor (from 0.915 to 0.916). However, this increase was not significant and both values reflect excellent reliability.^(25)^

Test-retest is often used to assess the reproducibility and stability of measurements. However, natural changes over the course of test-retest applications are thought to be the primary source of error in reproducibility estimation obtained using retest-based, particularly when intervals between applications are long or when maturational aspects interfere with test-retest results.^(28)^ In this study, this factor was minimized by test-retest application intervals of 1 week to 14 days. Also, reproducibility values (r) correlating both questionnaire application time points achieved good levels of agreement in all items (r>0.70).

Processes described in this study are only a part of the steps in a set of methodologies, which must encompass additional measurements to assess other types of validity and reliability. The Smoking-related Health Literacy Assessment Instrument instrument may provide researchers in this field with a user-friendly tool, with good levels of content validity and reliability, and applicable to longitudinal or interventional epidemiological studies in public and collective health, designed to investigate changes in smoking-related health literacy levels. Findings derived from the application of this tool in research settings can be used to inform related interventions.

## CONCLUSION

This study describes aspects involved in the process of creating and measuring of content validity and reliability of a scale aimed to assess health literacy about smoking. The EAS-T achieved good levels of validity and reliability. Test-retest revealed substantial to almost perfect levels of reliability (reproducibility).

Investigations with populations immersed in different contexts in the country are warranted to support findings of this study. Instrument development, including assessment of remaining psychometric properties, shall be continued.
